# Inevitability of the emergence and persistence of genetic parasites caused by evolutionary instability of parasite-free states

**DOI:** 10.1186/s13062-017-0202-5

**Published:** 2017-12-04

**Authors:** Eugene V. Koonin, Yuri I. Wolf, Mikhail I. Katsnelson

**Affiliations:** 1 National Center for Biotechnology Information, National Library of Medicine, National Institutes of Health, Bethesda, MD 20894 USA; 2 Institute for Molecules and Materials, Radboud University, 6525AJ Nijmegen, Netherlands

**Keywords:** Genetic parasites, Anti-parasite defense, Parasite-protected state, Thermodynamic instability, Replicators

## Abstract

**Abstract:**

Genetic parasites, including viruses and mobile genetic elements, are ubiquitous among cellular life forms, and moreover, are the most abundant biological entities on earth that harbor the bulk of the genetic diversity. Here we examine simple thought experiments to demonstrate that both the emergence of parasites in simple replicator systems and their persistence in evolving life forms are inevitable because the putative parasite-free states are evolutionarily unstable.

**Reviewers:**

This article has been reviewed by Yitzhak Pilpel, Bojan Zagrovic, and Eric van Nimwegen.

## Background

Nearly all cellular life forms are hosts to various types of genetic parasites that exploit functional systems of the host cells to replicate their own genomes [1]. Only some bacteria with highly reduced genomes that themselves lead a symbiotic or parasitic lifestyle seem to lack genetic parasites that undoubtedly have been lost during the reductive evolution of these bacteria from free-living ancestors. Genetic parasites include viruses, transposons, plasmids and other semi-autonomous genetic elements (SAGE) [2] that display a broad range of relationships with the hosts, from acute antagonism, whereby a virus rapidly kills the host, to symbiosis when SAGE are not costly to the host and could even have beneficial effects [3, 4].

Strikingly, virus particles appear to be the most common biological entities on earth. In most environments, the ratio between virus particles and cells varies between 10 and 100 [4–7]. This enormous physical abundance of viruses is matched by vast genetic diversity so that most of the gene repertoire of the biosphere appears to be concentrated in viruses, even as exact number remain a matter of debate [8–10]. The prevalence of viruses in the biosphere is also paralleled by the abundance of SAGE integrated in genomes of cellular life forms. Integrated SAGE are present in virtually all genomes of cellular organisms (again, missing only in some intracellular symbionts and parasites), and in genomes of multicellular eukaryotes, SAGE-derived sequences quantitatively dominate the genome, comprising at least 50% of the DNA in vertebrates and up to 90% in plants [11]. Recruitment of sequences from SAGE for cellular functions is a common phenomenon that made substantial contributions to the evolution of cellular life forms [12–14].

The entire course of the evolution of life is a history of host-parasite co-evolution [15–17]. Being subject to the constant onslaught of genetic parasites, cellular life forms have evolved a plethora of defense mechanisms. A typical organism harbors and interacts with multiple types of genetic parasites (e.g. viruses, different families of transposons, and plasmids) which it holds at bay thanks to multiple defense strategies that include parasite exclusion, innate immunity and adaptive immunity [18–23]. The SAGE respond with counter-defense mechanisms that range from simple mutational escape from defense to dedicated multigene systems that specifically inactivate host defense systems. Notably, defense systems and SAGE including their counter-defense machineries are tightly linked in evolution. Enzymes involved in the mobility of SAGE, in particular, transposons are often recruited by host defense systems for roles in parasite genome inactivation and other functions, and conversely, SAGE recruit components of defense systems that then evolve to become agents of counter-defense [24–26].

Thus, the arms race, along with cooperation, between genetic parasites and their hosts are perennial features of the evolution of life. Why is this the case? Why do the parasites emerge in the first place? And, could some cellular organisms actually get rid of the parasites through highly efficient defense systems? Empirically, the answer to the latter question seems to be negative. Conceivably, the general cause of the inability of the hosts to eliminate the genetic parasites is the unescapable cost of maintaining sufficiently powerful defense systems [27–30]. Analysis of theoretical models of parasite propagation suggests that an important source of this cost, perhaps the primary one in microbes, could be that efficient anti-parasite defense has the side effect of curtailing horizontal gene transfer (HGT), which is an essential process in microbial evolution that allows microbes to avoid deterioration via Muller’s ratchet [31, 32]. Another major factor could be the effectively unavoidable autoimmunity [29, 33, 34]. However, what about the first, arguably, the most fundamental question: why do genetic parasites evolve to begin with? Again, empirically, there is a strong impression that the emergence of such parasites is inevitable. Not only are they ubiquitous in cellular life forms but they also evolve in various computer simulations of replicator system evolution [35–39]. Furthermore, it appears intuitive: genetic parasites can be considered cheaters, in game-theoretical terms, and as soon as, in a replicator system, there is a distributable resource, such as a replicase, cheaters would emerge to steal that resource without producing their share of it [40]. These, however, are informal considerations. Here we ask the question: is it possible to develop a theoretical framework that would allow a formal demonstration of the inevitability of the emergence of genetic parasites in evolving replicator systems, or else, that parasite-free replicator systems are after all possible?

### Evolutionary instability of parasite-protected replicators

**Fig. 1:**
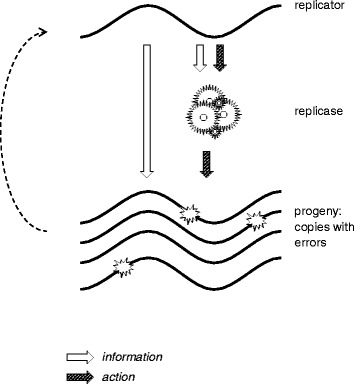
A replicator-replicase systems with heredity, variability and differential reproduction. The *dotted arrow* denotes differential reproduction of the copies of the original replicator that carry mutations

Let us try, as a Gedanken experiment, to construct a self-replicating entity that is strictly resistant to parasites. Consider a simple system consisting of a replicator, serving as a template for itself, and the replicase it encodes (Fig. 1). The replicator is assumed to contain the replicase-encoding signal (RES) (the replicase could be a protein, a ribozyme, under the RNA World model, or, in theory, any other entity capable of catalyzing replication of a template) and the replicase recognition signal (RRS). Although we attempt to make this discussion as general as possible, it seems relevant to note here that, at least, in extant replicator systems, the signals that ensure the specificity of replication are relatively simple and are much smaller than protein-coding genes [41, 42].

Evolvability is a fundamental and inescapable property of such a replicator-replicase system [43]. To be evolvable, a system must possess three basic properties: 1) heredity (whereby the location of progeny in the phenotype space is correlated with that of the parents), 2) variability (whereby the progeny is not identical to the parents), and 3) differential reproduction (whereby the capability of a replicator to leave progeny is part of the phenotype). Heredity is ensured by replication with fidelity above the error catastrophe threshold. The replicator theory that was developed primarily by Eigen and colleagues demonstrates that, under simple fitness landscapes, there exists a replication fidelity threshold, below which the master sequence in a population of replicators cannot be efficiently passed across generations, so that the entire population collapses [44, 45]. Elucidation of the molecular mechanisms of primordial replication that could provide for crossing the error catastrophe threshold remains a daunting task that is central to the entire origin of life field. However, for the purpose of the present discussion, we assume that a sustainable replicator system with a minimally acceptable fidelity has evolved.

Variability is ensured because, at any temperature above 0 K, any process is subject to entropy-increasing fluctuations and, therefore, replication is inherently error-prone, under the second and third laws of thermodynamics, and given a finite energy supply.

Differential reproduction ensues from the fact that the replicator encodes the replicase that, in turn, copies the replicator itself. Mutations in both RES and RRS can affect the efficiency of replication.

If the resources that are available to the system are limited (i.e. the system cannot support unlimited growth of all possible constituent parts), competition between individual replicators ensues and selection arises. In a system with finite memory storage, all information exchange, transfer and utilization processes carry a memory clearing cost of at least *kT*ln2 J/bit, where *k* is Boltzmann constant and *T* is temperature. The existence and value of this minimum information cost is known as Landauer’s principle [46] which is a corollary of the second law of thermodynamics, under the information-theoretic interpretation of thermodynamics [47–49] In all known systems, this cost is many orders of magnitude higher [46, 48, 49]. Therefore, selection for cost reduction acts not only on the constituent parts of the system, but also on the information transfer processes themselves, effectively ensuring an upper limit on the fidelity of information transmission.

Selection acts on both RES (eliminating replicators encoding inefficient replicases) and RRS (eliminating replicators that are inefficient as templates, e.g. are poor replicase-binders), but these two selection processes act on the replicator through physically different agents (the replicator-encoded replicase and the replicator itself, respectively).

**Fig. 2:**
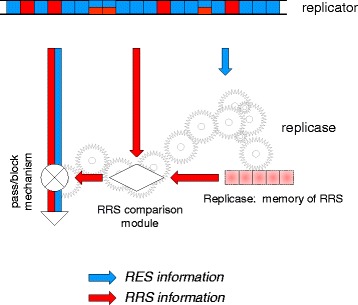
The replicase-encoding signal (RES) and replicase-recognition signal (RRS) in replicator-replicase systems. For generality, the RRS is shown as being distributed along the length of the replicator although in real genomes, this signal is often localized such that, for example, short terminal sequences are sufficient for the replication of a virus genome. The replicase structure carries memory of the RRS allowing recognition of competent templates (“pass/block mechanism”)

The dual nature of the replicator (acting as both the template and, directly or indirectly, as the replicase) necessitates that the information embedded in the RES and RRS is realized via physically different processes. The RES guides the formation of the replicase which, in turn, recognizes the RRS. Such recognition implies comparing the RRS in the replicator with some form of memory encoded in or attached to the replicase (Fig. 2).

**Fig. 3:**
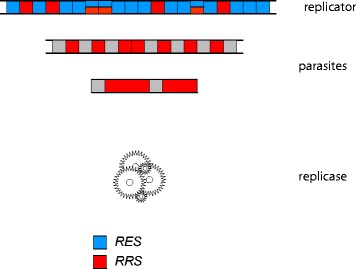
Emergence of parasites in replicator systems via deletion of portion of the RES

A general, simple way of parasite emergence involves skipping part of the RES during replication, resulting in a shorter replicator that consists of the RRS and, in the extreme, nothing else (Fig. 3). This straightforward mechanism for parasite evolution is inspired by and is similar to the process of RNA shrinking that was observed during in vitro evolution in the classic early experiments of Spiegelman and colleagues [50–52].

**Fig. 4:**
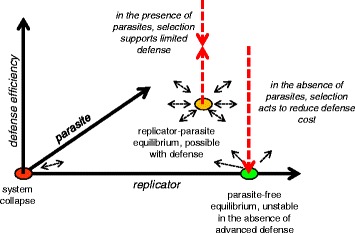
A conceptual phase diagram of the evolution of replicator systems

Under the scheme in Fig. 3, parasites emerge as long as the information content of the RRS is less than that in the full replicator, i.e. when the RRS is at least partially separable from the RES. If this is the case, a replicator containing the full RRS, but omitting at least some of the RES (RRS_p_ ≡ RRS, RES_p_ - > 0; the subscript ‘p’ denotes the respective signals in the parasite), would not only serve as a template as efficient as the original replicator, but would also enjoy an evolutionary advantage because replication of the smaller replicator is faster and requires less resources (building blocks, such as nucleotides, and energy). This makes the parasite-free equilibrium point of the replicator-parasite system unstable because deletion of any part of the RES yields more efficient replicators (Fig. 4). Therefore, the system is vulnerable to parasite invasion, and moreover, such an invasion is inevitable under a non-zero parasite emergence rate (see Appendix for a more formal demonstration).

It appears that, under this scheme, the only way to render the replicase-producing replicator parasite-protected is to make the RRS to include the entire RES (Figs. 2 and 4). Such RRS ≡ RES configuration evidently rules out the emergence of a parasite because any mutation of the RES would also inactivate the RRS and prevent replication.

However, such a parasite-protected state is subject to the aforementioned instability (Fig. 4). In the absence of parasites, perfect protection does not carry any benefits, but incurs a greater cost than less protected states. Given that the system is evolvable, an RRS < RES state will inevitably arise and outcompete the RRS ≡ RES progenitors that are, as shown above, prone to emergence of genetic parasites (RRS_p_ ≡ RRS, RES_p_ - > 0).

From a more abstract perspective, the fully protected RRS ≡ RES system corresponds to the maximally constrained, i.e. minimum entropy, state. The second law of thermodynamics (once again, under the assumption of a limited energy supply) effectively guarantees that it evolves into a higher entropy state, such that RRS < RES, and at least some parts of the RES can be mutated or deleted without compromising replication. An additional important and highly plausible assumption predicating this conclusion is that the entire replicator system is stable on the time scale of RRS evolution, and accordingly, the evolving system can be considered to be at quasi-equilibrium. The ensemble of higher entropy states is obviously more robust than the unique RRS ≡ RES state. In biological parlance, the higher entropy states are favored by selection via the ‘survival of the flattest’ route [53]. They will necessarily prevail because there are plenty of such states with similar fitness values, whereas the RRS ≡ RES state is singular. However, the problem with the ‘relaxed’ states of the replicator is that they are no longer protected from parasites because a parasite now can evolve that would exploit the RRS without producing the replicase (Fig. 3). The third law of thermodynamics dictates that the minimum (zero) entropy state can be stable only at 0 K. Under the detailed correspondence between thermodynamics and population genetics [54], the equivalent of temperature is the inverse effective population size, and accordingly, 0 K corresponds to an infinite population, which can exist only as an abstraction. Thus, the two complementary phenomena, i.e. microscopic replication errors, that are statistically inevitable at a non-zero physical temperature, and fixation of neutral or slightly deleterious mutations in a population, which in its turn is statistically inevitable in a finite population, jointly ensure that a parasite-free replicator system is inherently unstable and either goes extinct or rapidly spawns parasites (Fig. 4). The latter case inevitably triggers the host-parasite arms race, whereby in the simplest case, the hosts evolve by selection for changed RRS allowing them to escape the parasites, whereas the parasites catch up. Furthermore, the competition also occurs between the parasites themselves and could eventually result in the emergence of ultimate parasites, those that consist entirely of the RRS. Such (near) ultimate parasites were the end result of Spiegelman’s experiments [52] and also exist in nature, namely the viroids, small, non-coding parasitic RNAs that cause disease in plants and rely entirely on a host-derived replication machinery [55, 56].

It should be noted that, because, at least in the simplest replicator system, the replication rate is inversely proportional to the genome length, the parasites have an intrinsic advantage in the arms race. In the well-mixed case, the preferential replication of parasites drives the host to extinction which, obviously, results in the collapse of the entire system (no replicase is produced anymore).However, spatial heterogeneity (compartmentalization) can stabilize host-parasite systems even in the absence of active defense. Thus, parasites drive evolution of biological complexity [37–39, 57].

There seems to be a symmetry between the hypothetical, minimum entropy, parasite-resistant replicator and the ultimate parasite with RRS_p_ ≡ RRS, RES_p_ ≡ 0, another minimum entropy state, this one being the theoretical end result of the competition between parasites (Fig. 3). As a minimum entropy state, the ultimate parasite cannot be evolutionarily stable either. The Gedanken experiment described above certainly is an idealization. Realistically, when the minimum entropy state relaxes, the host-parasite arms race takes more complex forms. Most parasites are far from this ultimate state but rather possess a number of genes and encode a variety of functions. This complexity of parasites has to do with two strategies that parasites evolve to maximize their evolutionary success, namely: 1) overcoming the defense systems which the hosts evolve under the pressure for resistance to the parasites, and 2) surviving outside the host and disseminating among hosts [4]. The question, then, emerges: even though the above analysis shows that evolution of parasites in simple replicator systems is inevitable, is there a chance that evolution of defense systems would exterminate parasites?

### Costs and compromises of anti-parasite defense

The thought experiment described above also answers the question whether a perfect defense system can exist. A perfect self vs non-self discrimination, whereby a replicator possesses the means to reject or destroy any potential cheater, that is, any sequence other than a perfect copy of itself, is nothing but the same parasite-protected system with recognition based on the complete information on the self, i.e. RRS ≡ RES. We have already shown above that such a system is evolutionary unstable from pure thermodynamic considerations because it provides no benefit in the absence of parasites, and will inevitably devolve to an RRS < RES configuration (“leaky” defense).

The existence of an unavoidable cost implies that maintenance of any form of defense is subject to a cost-benefit tradeoff. Notably, a recent quantitative assessment of the selection coefficients (a measure of fitness cost) associated with different classes of genes in microbial genomes has shown that defense systems are as costly as the more benign SAGEs, such as transposons [30]. This result seems to be a genome-scale reflection of the intrinsic costliness of defense dictated by thermodynamics. In the actual biological context, this cost can be manifested in different forms, such as autoimmunity or interference of defense systems with HGT. The curtailment of HGT, in particular, could be a key factor that makes defense systems costly and causes their repeated loss [32]. However, at the bottom of it all seems to be the thermodynamic cost of information. Certainly, defense is not the only process associated with an information cost. Such cost is intrinsic to all processes of information transmission including replication, transcription, translation, as well as signal transduction. However, loss of genes encoding those other functions is often strongly deleterious to the organism, resulting in positive mean selection coefficients for the respective classes of genes [30]. This is not the case for defense systems: the mean selection coefficient values for the defense genes are negative [30], and in accord with this observation, defense genes are lost in the course of evolution significantly more often than genes of other functional categories [58]. Due to these fitness costs, evolving organisms cannot build up defense systems to the extent that is required to eliminate parasites (Fig. 4).

### A quasi-formal demonstration of the inevitability of the emergence and persistence of parasites

In the above, we demonstrate that a parasite-protected state, one in which RRS ≡ RES, is thermodynamically unstable. The inevitability of the emergence of parasites and their subsequent persistence follows from this demonstration. To present the argument succinctly and quasi-formally:

1. The two signals that are essential in a replicator system, RRS and RES, are encoded in different genomic sequences.
2. Thus, at least some parts of the RES can be deleted without inactivating the RRS. Hence genetic parasites emerge.
3. The shorter the genome sequence of a genetic element the more efficient its replication is. Hence parasites accumulate in a replicator system and may bring it to collapse in a well-mixed case.
4. A perfect defense system should, in the least, be able to recognize parasitic elements, i.e. detect missing parts of the host genome. At the end of the replication cycle, this information should be deleted. Under the Landauer principle, the cost of memory cleaning is at least *kTn*ln2 J (*n* bits distinguish the host and the parasite). This cost makes defense systems evolutionarily unstable and precludes elimination of parasites.

## Conclusions

The problem of the ubiquity and persistence of genomic parasites throughout the evolution of life can be broken into two parts: i) emergence of parasites in primitive replicator systems, ii) persistence of parasites in evolving organisms. Our analysis of the basic aspects of host-parasite coevolution presented here suggests that there are fundamental thermodynamic causes of the inevitability of parasites on both stages. To put it most succinctly, both the hypothetical parasite-protected state in a simple replicator system and the putative secondary parasite-free state resulting from efficient action of advanced defense systems are evolutionarily unstable and can only exist transiently. These conclusions certainly are compatible with a wealth of observational data indicating that genetic parasites, along with defense systems, are enormously abundant in the biosphere and accompany virtually every cellular life form. The results of numerous mathematical and computational modeling studies on replicator evolution, in which parasites invariably appear, lead to the same conclusion. The simple thought experiments described here that start, effectively, from first principles emphasize the growing understanding that emergence as well as persistence of genetic parasites is an inalienable feature of evolving replicators and, as such, one of the central principles of biology.

## Appendix a

Consider a simple system in which both the replicator and a parasite comprise single entities and the replicator entity can also function as the replicase (e.g., both the replicator and the parasite are single RNA molecules, and the replicator molecule in the native folded state can function as the replicase ribozyme). Replication of the replicator entity is governed by the second-order kinetics, where a template replicator meets an (identical) replicase to make a copy of the template. There is also a natural decay of both types of entities, occurring with the first-order kinetics. In an environment with a fixed carrying capacity (determined, e.g., by the influx of consumable resources), such a system behaves as a classical logistic model, with one exception: there exists a critical number of entities, below which the population is unsustainable due to the (second-order) replication lagging behind the (first-order) decay.

Likewise, a parasite entity replicates upon meeting a replicator (acting as a replicase) and decays spontaneously. The parasite population is subject to similar environmental restrictions as that of the replicator due to the limitation by the same resources.

The general model of evolution employed here is derived from the classic logistic equation [59], $\frac{dX}{dt}= rX\left(1-\frac{X}{K}\right)$, where *X* is the population size, *r* is the growth rate and *K* is the population carrying capacity. Under the logistic model, the growth is limited by utilization of available resources influx by the growing population; the equilibrium is reached at *X* = *K*.

For the purposes of this work, we modify the logistic model in the following way. First, we consider separately the populations of the bona fide replicators and the parasites (*R* and *P* respectively). Then, we explicitly introduce the death process at the rate proportional to the population size (*e*_*R*_ *R* and *e*_*P*_ *P*) and the second order kinetics of replication, dependent on the interaction of the replicator and the template (*R* ^2^ for the replicator and *RP* for the parasite). The replicator and the parasite utilize the same resources (the latter being more economical by a factor of *q*), so both contribute to the numerator above the carrying capacity (*R* + *P*/*q*); also, the parasite replicates faster than the replicator by the same factor *q* (the simple conceptual model of this effect is based on an RNA molecule that is shorter than the replicator by a factor of *q*). Finally, the existence of a (potentially costly) defense system, embedded in the replicase, is taken into account in the form of a coefficient, dependent on the defense systems efficiency e*. The action of the system inhibits the growth of the parasite but incurs a cost to the replicator that is also dependent on e* with the cost coefficient α (according to the Landauer principle, α > 0):

$$ {\displaystyle \begin{array}{c}\frac{dR}{dt}=\frac{1}{1+\alpha {e}^{\ast }}{R}^2\left(1-\frac{R+P/q}{K}\right)-{e}_RR\\ {}\frac{dP}{dt}=\frac{q}{1+{e}^{\ast }} PR\left(1-\frac{R+P/q}{K}\right)-{e}_PP\end{array}} $$

(the replicator growth rate is taken to be 1 without loss of generality).

Upon introduction of the parasite to the population of the replicator near the equilibrium (*P* → 0, *dR*/*dt* → 0), the host defense prevents the parasite invasion (*dP*/*dt* < 0) only if

$$ \frac{1+{e}^{\ast }}{1+\alpha {e}^{\ast }}>q\frac{e_R}{e_P}\cong q $$

or, in other words, only if the effect of defense on the invading parasite relative to the cost of defense to the host is greater than the parasite advantage *q* (assuming comparable decay rates).

Obviously, the replicator population lacking the defense system (e* = 0) cannot resist the parasite invasion. A defense system combining high efficiency with low cost is capable of protecting the host population, but the maintenance of such defense depends on regular invasion of parasites (otherwise, the evolutionary disadvantage due to the cost of defense would drive the defense system to extinction). In other words, a parasite-protected state of replicator system is unstable.

## Reviewers’ reports

### Reviewer 1: Yitzhak Pilpel (Weizmann Institute of Science)

This is a very elegant argument that indirectly explains the common observation that essentially all non-parasitic life forms are subject to parasites. The argument is valid, the model is adequate and the paper is very well written. As such it will make a nice contribution to the field, and publication is Biology Direct is certainly recommended.

This paper, on the inevitability of parasites in biological systems, provides a thermodynamics – based formal argument that parasitic (viral like) systems are bound to emerge and persist in any biological system. As ‘biological systems’, the authors consider a simplest cases of a replicator that is encoded from a ‘genome’ which consists of a region that encodes for the replicase (RES), and a recognition site within that genome (RRS) that is to be recognized by the replicase to ensure replication. A parasite can emerge as a variant of the replicator which omits at least part of the RES, thus saving replicase expenses, but that nonetheless preserves the RRS to a sufficient extent such that it may still get replication services from the host replicase. A key to the argument is the host’s defense system that, if abstracted appropriately as done here, can be thought of as means for a host replicator system, to discriminate against a parasite RRS’s in favor of the host’s, in a replication cycle, yet at the investment of costly resources. The question then becomes, how stable is a system that consists only of a host replicator without a parasite, e.g. in the face of ‘invasion’ of a parasite, or simply when host replicators, inevitably mutate into parasites. The argument, presented as a thought experiment, and also as a mathematical formal model is simple: the hosts will sustain the costly defense system, provided that it is sufficiently discriminative in its favor, provided that the parasite emerges frequently enough. If the parasite is defeated the host will lose the defense system, and will hence become sensitive to a future attack. This means that the parasite free system was instable. This is a very elegant argument that indirectly explains the common observation that essentially all non-parasitic life forms are subject to parasites. The argument is valid, the model is adequate and the paper is very well written. As such it will make a nice contribution to the field, and publication is Biology Direct is certainly recommended.

Response: *We appreciate the constructive assessment of our work*.

I do have comments and suggestions though. 1. The potential protected state defense “RES = RRS”, is shown to be instable due to cost considerations. Yet some potential embodiments of the RES = RRS solution could have little or no costs. For example, in real-genomics terms, if the origin of replication of the host was embedded within the sequence encoding for the DNA replication machinery, protection could be provided with no cost. Could the authors comment on this possibility? I’m wondering if real genomes feature such a solution.

Response: *Actually, as shown in* Fig. 2 *, we explicitly consider the possibility that the RES and the RRS overlap. A protected state emerges only when RES ≡ RRS, and this state is inherently unstable.*

2. Can the model account for “nested parasitism”, i.e. a parasite of a parasite. For example consider the case of a SINE and a LINE, in which the SINE could limit the spread of a LINE – what are the stable solutions of a three-way system that consists of a host, primary parasite and a secondary one?

Response: *nested parasitism is indeed a very interesting phenomenon that has been explored using mathematical models as well as experimentally, in particular, for the virus-virophage-host systems. However, this is beyond the scope of the present analysis that deliberately focuses on the most basic host-parasite systems.*

3. The model (esp as stated explicitly in its math embodiment) assumes that the only way through which the parasite inhibits the replicator host is due to their sharing the growth resources ((R + P/q)K), while in reality some parasites such as viruses can inflict more severe damage. I wonder if effects such as lytic infection should be modeled explicitly.

Response: *Once again, we consider the most fundamental host-parasite systems in which the damage inflicted is minimal,* i.e. *indeed limited to resource appropriation. In biological terms, this corresponds to the first replicators, supposedly, within the hypothetical primordial RNA world. The lytic viruses as well as symbiotic plasmids are products of subsequent evolution. The diversification of parasite strategies is an important subject for theoretical study that we expect develop in the future but not in this paper*.

4. Regarding the mathematical model of Appendix A, a few comments. (i), it is not clear to me why the discrimination efficiency factor e* appears identically on the replication and parasite eq. I’d imagine it appearing only on the parasite, and depending on its exact definition (which is missing), perhaps appearing as 1-e* in the replicator equation. (ii), I’d recommend for the non-math oriented reader to introduce first the basic logistic model (dx/dt = rx(1-x/k)-ex). (iii) the steady state stability analysis is not provided in a rigorous fashion, if the authors go through presenting the ODEs they could perhaps do the extra step of formally identifying all the steady state and analyze their stability. (iv) related – it was not clear to me if Fig. 4 represents such stability analysis, that is directly based on the equations, or is it hand drawn result of the thought experiment.

Response: (i) Actually, the discrimination efficiency factor e* does not appear identically in the equations for the replicator dynamics and the parasite dynamics: in the former, it appears with the cost coefficient α. Less formally, we chose a simple generic function which is required to have the following properties: defense reduces the replications rates of both the host replicator and the parasite, and the magnitude of this effect is co-monotonic with e*; (ii) In the revised version of the Appendix, we follow this suggestion; (iii) we believe that the simple analysis described in the paper is sufficient to demonstrate the instability of the parasite-protected state in the analyzed toy model; complete analysis of phase portraits in more complex systems is beyond the scope of the paper; (iv) Fig. 4 *is indeed a hand drawn results of the thought experiment (see point iii).*

### Reviewer 2: Bojan Zagrovic, University of Vienna

The authors provide a novel and potentially far-reaching, albeit largely qualitative argument concerning the thermodynamic necessity of genetic parasite evolution i.e. instability of parasite-free replicator systems. Given the complexity of the problem at hand and the simplicity and the generality of the proposed framework aimed at addressing it, there by necessity exists a degree of vagueness in the abstract arguments presented, but this I do not hold against the authors. They are attacking a fundamental question and their arguments involve only the fundamental tools e.g. the second law of thermodynamics - this generality is in fact the main strength of the article, which makes it worthy of being published, studied and potentially extended.

Response: *we appreciate the constructive assessment of our work*.

While I found the article stimulating, my main concerns relate to the difficulty of understanding the thermodynamic aspects of the authors’ arguments. Specifically, the authors interchangeably refer to thermodynamics in a classical, physical sense and, at the same time, in the context of a previously demonstrated analogy with population genetics formalisms. For example, the term temperature is used both in its standard, physical sense and in a sense of population genetics, where zero temperature refers to populations of infinite size. Similarly, when they speak about, they refer to free energy cost of memory clearing in its canonical sense, while on the other hand it is not clear what this would mean in the population genetics sense. This dual usage adds to the difficulty of following and fully appreciating the main argument. It would be beneficial if the authors devoted more space to clarifying the distinction between the two.

Response: *we believe that both the traditional thermodynamic perspective and its counterpart in population genetics are relevant and important. To connect the two, we added the following in the revised manuscript*: “Thus, the two complementary phenomena, i.e. microscopic replication errors, that are statistically inevitable at a non-zero physical temperature, and fixation of neutral or slightly deleterious mutations in a population, which in its turn is statistically inevitable in a finite population, jointly ensure that a parasite-free replicator system is inherently unstable and either goes extinct or rapidly spawns parasites (Fig. 4).”

On the other hand, I have found the authors’ arguments concerning a “perfect” defense system in which RES = RRS to be very stimulating and potentially relevant in the context of another question of a wide biological significance, that is, the origin of the universal genetic code. Namely, among different hypotheses, the stereochemical hypothesis of the code’s origin has received significant attention over the past decades [60, 61]. Specifically, the hypothesis suggests that the code evolved from direct interaction preferences between amino acids and their cognate codons. Recently, we have generalized this hypothesis to suggest that if amino acids should interact specifically with their cognate codons, then oligopeptides should also interact with their cognate mRNAs in a complementary, co-aligned fashion, especially if unstructured [62–64]. In fact, we could demonstrate that the nucleobase-density profiles of modern day mRNA coding sequences exhibit a close matching with the corresponding nucleobase affinity profiles of their cognate protein sequences, giving support to the generalized version of the stereochemical hypothesis. Now, a putative primordial scenario in which such complementary interactions could have taken place is one in which a given mRNA encodes a corresponding protein, but also directly interacts with it (mRNA as a direct template for synthesis of a protein). If we envision that the protein in question is the ancient replicase and that the two interact along the whole sequence, then we have a situation in which RES = RRS. In other words, it appears that the generalized version of the stereochemical hypothesis may actually be consistent with a picture of a replicator with a perfect (or, at least, strong) defense system against parasitic genetic elements, as discussed by Koonin et al. As the authors discuss, such a perfect system may be unstable, but this still allows for a possibility that it serves as a useful asymptote towards which evolution might gravitate towards. What I then find particularly exciting is the possibility that such a drive towards a high-level (although perhaps not perfect) discrimination between hosts and parasites might have led to the development of the universal genetic code as we know it. In other words, it is possible that the code is as it is, in part because of an attempted optimization of high-level host-parasite discrimination. I wonder if the authors would care to comment on this possibility and perhaps develop it even further. To the best of my knowledge, I have never encountered any similar discussion linking the origin of the genetic code with the evolution of the host-parasite relationship.

Response: *this is definitely an interesting line of discussion but in the context of the current article, we address a more coarse-grained model to address the basic principles of parasite emergence and evolution*.

1. “gedunken experiment” should be spelled “Gedanken experiment”.

Response: *we regret the error; corrected*.

## Reviewer 3: Eric van Nimwegen, Biozentrum, Basel

This is an interesting paper for people interested in the evolution of parasites. While the general evolutionary arguments are valid, the authors also suggest in various places that there is some more fundamental basis for their arguments rooted in thermodynamics and those arguments are really just wrong and based on a misunderstanding of the physics.

Response: *We appreciate both the general positive assessment and the criticism. Below, however, we argue that the thermodynamic considerations are relevant and correct, after appropriate modification*.

The authors present a number of arguments and gedanken (not ‘gedunken’ as was written in the manuscript) experiments for why the emergence of parasites is virtually guaranteed within systems of replicators. I generally agree with the authors that situations without parasites are very hard to stabilize from an evolutionary perspective, and the authors do a reasonably good job of giving some insightful arguments for why this is. This is especially useful to make clear that one doesn’t need special explanations for why arms races between replicators and their parasites persist in evolution. Those parts of the paper I liked and agree with. There’s two things that I didn’t agree with. First, the writing suggests that the authors believe their arguments almost amount to something like a mathematical proof that situations without parasites can never be evolutionarily stable. I don’t believe this is correct. I don’t believe that the arguments in the paper have really exhaustively covered any possible conceivable replicator system, i.e. I simply do not believe that we have as much imagination as nature might have in this respect. Moreover, even for the situations discussed in the paper, I can think of some caveats that could threaten the argument (mentioned below). Even if I don’t think these are likely to occur in practice, they do affect the ’mathematical rigor’ of the argument. This criticism can be easily addressed by toning down the language regarding how definitive the arguments are that are being presented.

Response: *Certainly, the model presented in the paper does not exhaust all conceivable replicator systems. There is no claim of such universality in the article. However, we do submit that the model represents the most basic replicator system readily imaginable. We explicitly state the assumptions under which the model is applicable (these assumptions have been clarified in the revision as indicated below). We do not claim a mathematical proof of inevitability of parasites (which is impossible by definition) but we do believe we present strong arguments rooted in fundamental physical principles.*

The parts of the paper that I have most trouble with concern the attempts to make connections between what are essentially arguments about evolutionary dynamics, and notions from thermodynamics, including the second and third laws, and Landauer’s principle. I strongly feel that these thermodynamic considerations have nothing whatsoever to do with the topic of this paper and I believe the paper would be much better if all these references to thermodynamics were removed. I know that there is an enormous amount of confusion and misleading arguments in the literature around the topics of the second law, the arrow of time, the relation between thermodynamics and computation, and the relation between thermodynamics and biological evolution. I get the impression that the authors have fallen prey to some of these misleading arguments. The issues are technically simple but conceptually full of pitfalls and are really beyond the scope of a small review like this. I will just make a few remarks and point to literature that I find relevant. First, regarding the connections between population genetics and thermodynamics. This is really nothing but the appearance of similar mathematics in different subject areas. The reason that such similar mathematics appears is because the maximum-entropy methods of statistical physics are general methods of statistical inference, that will apply in many situations, including population genetics. I refer the authors to the work of E.T. Jaynes, who has explained this most clearly in my opinion (see, e.g., chapter 11 of ‘probability theory, the logic of science’). The relations between the second law, irreversibility, and computation all ultimately stem from the fact that the laws of physics are reversible at the microscopic level (note that we are here not concerned with quantum measurement, collapse of wave-functions, or many universes splitting, etcetera. That’s conceptually an even much bigger can of worms that I would prefer to keep closed here). Because of this microscopic reversibility, Liouville’s theorem guarantees that any collection of states in microscopic state space retains its volume under time dynamics. One can think of a thermodynamic ’macrostate’ as nothing but a particular collection of microscopic states and the entropy of this macrostate essentially corresponds to the logarithm of its volume. Since volume is conserved, it is impossible for a macrostate to evolve into another macrostate with LESS volume, i.e. entropy cannot go down. This is the essence of the second law. (See, for example, Jaynes, E. T., 1965, ’Gibbs vs Boltzmann Entropies,’ Am. J. Phys., 33, 391 which, in addition to many other conceptually enlightening papers can be downloaded from http://bayes.wustl.edu/etj/node1.html). Similarly, whenever a subsystem undergoes some dynamics that is irreversible in the sense that multiple input states map to the same output state, this corresponds to a contraction in the volume of microstates of this subsystem. Since the microscopic laws are time reversible, the joint volume in state space for the subsystem plus its environment must be conserved, and thus a contraction in volume for the subsystem must be accompanied by an expansion of the volume of the environment. By the definition of entropy, this expansion corresponds to an increase in entropy. This is the reason why all systems that sustain irreversible behavior must be dissipating entropy (and, at finite temperature kT times as much energy) to their environment. Landauer’s principle is just one example of this, ie. erasing memory is irreversible. However, none of this has much of anything to do with the evolution of replicators and their parasites.

Response: *as it could be suspected, the authors are familiar with the work of Jaynes and related literature. There is no argument at all regarding the validity of these concepts and theory. What we fail to see, is why Landauer principle is irrelevant when it comes to the evolution of replicators and their parasites. It defines the low bound on the energy cost and hence constrains evolution. In the revision, we specifically refer to the information-theoretic interpretation of thermodynamics and cite Jaynes’ book.*

The key point is that any replicating system is constantly performing irreversible operations (e.g. when it is copying itself) and dissipating energy in the process. Arguing against defense systems because they require energy dissipation seems fundamentally misguided to me because energy dissipation is what replicating systems do by definition (i.e. take energy sources from the environment and turn it into copies of themselves). There’s no reason to believe that evolution would act to minimize energy dissipation in their functioning. In fact, it’s probably more accurate to think of evolution as a competition between replicators for dissipating energy from the environment as fast as possible, i.e. faster replicating cells are dissipating energy from the environment faster. I realize this is a fundamental discussion that is much beyond the scope of this review but I have really no doubt that the viewpoint that the authors present is simply incorrect on the connection between evolution and physics, and I think it is really unhelpful to further add to the confusion on this topic. I feel the evolutionary arguments are valid without any appeal to thermodynamics and the paper would be much improved if thermodynamics (and Landauer’s principle) were not mentioned at all.

Response: *This is a very interesting comment where we seem to disagree with the reviewer on substance. We think there is every reason to believe that, in a finite resource situation, selection does act to minimize energy dissipation by evolving replicators. Actually, the statement that replicators evolve to dissipate energy as fast as possible is outright false in its simplest form. Fast replication leads to an increased error rate and eventually to collapse of any replicator systems. Hence the trade-off between replication rate and accuracy that can lead to different optimal solutions during evolution. Evolutionary success of many fastidious organisms is broadly recognized, and in spatially organized environments slow-growing strains actually systematically outcompete fast-growing ones* [65, 66].

Regarding ‘adding to the confusion’, we certainly must avoid this. In the revision, we emphasize in several places that we address here energy costs under the limited resources assumption not simple application of laws of thermodynamics under equilibrium. With these qualifications, we believe that the physical considerations here are relevant and even essential*.*

Some more detailed comments: page 5, lines 98-100: “replication is inherently error prone” I believe this is a misleading statement. As Hopfield’s kinetic proofreading principle has shown, one can use dissipation of energy to make copying as accurately as one wants. There’s a whole field on error-correction mechanisms in computation that show that, if one is willing to pay energy, there are no fundamental bounds on the accuracy that can be achieved. Page 5, line 110: “in all known systems, this cost is many orders of magnitude higher” I do not understand what ‘this cost’ is referring to here.

Response: *We appreciate the comment. Once again, the quoted statement required a crucial qualification that replication is error-prone given a finite energy resource which, obviously, is the case in any realistic situation. There is no doubt that dissipation of energy can be harnessed to achieve any desired accuracy of copying but the problem is the energy cost of such solutions*. *Such qualification was added in the revised manuscript*.

In any case, as I have argued above, I strongly feel Landauer’s principle is irrelevant for the problem of evolutionary stability of parasite-free states. Page 6, lines 112-113: Yes, cost concerns may in principle put a bound on how much energy an organism is willing to spend on accuracy of replication. But I wonder to what extent this plays a role in practice. For example, evolution has led *E. coli* to evolve its replication error down to something like 2 * 10^(−10) errors per base, i.e. about 0.001 an error per entire genome replication so that only one in every 1000 divisions leads to ANY error made in the copying. If replication accuracy is as costly as the authors seem to suggest, what selection pressures do the authors imagine could have led *E. coli* to make its error rate so low (keeping in mind that a large fraction of mutations are likely selectively neutral)? It seems that either we have to assume that even one point mutation in 1000 genome replications is still very costly from the point of view of fitness (which I find hard to imagine), or we must conclude that the cost of accuracy in replication is apparently not really that large in realistic settings.

Response: *E. coli or any extant cellular life form is a product of > 3 billion years of evolution during which the error rates have been optimized through the evolution of diverse repair systems, under the pressure of different evolutionary factors, one of the most important being the continuous parasite pressure. That said, the high fidelity of replication does not at all preclude evolution extensive gene loss* via *recombination that results, in particular, in the evolution of parasites with minimal genomes* [67]*,* e.g. *intracellular bacteria, such as, for instance, Buchnera, a close relative of E. coli* [68].

page 7, lines 141-147: One loophole I can see here is that it seems to be assumed that the genotype-phenotype mapping is continuous in the sense that different functional RES solutions not only exist, but are reachable through point mutations. It is conceivable that many RES solutions exist, but that they are like ‘single spikes’ in genotype space so that it is virtually impossible to mutate from one functional to another functional RES.

Response: *We believe that there is a typo in this comment,* i.e. *the reviewer means RRS not RES. Under this understanding, we indeed assume smooth genotype-phenotype mapping whereby small changes (not necessarily point mutations) can have non-negligible effect on the efficiency of the RRS. To the best of our understanding, this assumption is realistic given the relative simplicity and high plasticity of the replication signals. We added a comment to this effect in the revised manuscript.*

page 7, line 150: ‘effectively guarantees that it evolves into a higher entropy state’ This is just wrong. We are in a non-equilibrium situation where replication is itself driven by energy dissipation and the second law gives no guarantee whatsoever about which way the evolutionary process will go in this situation.

Response: *This is a relevant comment, and in response, we added the following to the revised text:* “An additional important and highly plausible assumption predicating this conclusion is that the entire replicator system is stable on the time scale of RRS evolution, and accordingly, the evolving system can be considered to be at quasi-equilibrium.”

page 7, lines 153-154: ‘survival of the flattest route [50]’. I cannot help myself from mentioning here that this work largely revisits theoretical results that were first published in [69]. I can see another possible loophole here (apart from the appeal to thermodynamics which, again, I am convinced is mistaken). I think it would be possible to have an evolutionarily stable system in which there is co-existence of a whole ’quasispecies’ of replicators and parasites, which includes SOME replicators that are parasite-free, and some that are not. That is, while there is no benefit for a defense system in the absence of parasites, there IS when there are parasites around and I believe one could have a stable quasispecies-like state in which there are stable ‘wild types’ with defense systems that protect them from parasites, that co-existence with ‘mutant’ forms in which the defense system works imperfectly and that ARE infected by parasites, which are maintained by exploiting these mutants. Thus, I believe one could have an evolutionarily stable system in which SOME of the organisms are perfectly defended against parasites.

Response: *These are interesting possibilities but the system described by the reviewer is highly complex, and as far as we can see, there is no evidence of its plausibility*.

page 9, lines 221-222: “is thermodynamically unstable” No. The thermodynamic arguments do not apply to the non-equilibrium situation of the replicators.

Response: *As discussed above, the situation can be considered quasi-equilibrium, and the thermodynamic considerations apply*. *Nevertheless, we toned it down somewhat by replacing “thermodynamic instability” with the less restrictive “evolutionarily instability” in several places including the title and the abstract.*

## Abbreviations

HGT: horizontal gene transfer
RES: replicase expression signal
RRS: replicase recognition signal
SAGE: semi-autonomous genetic elements
